# A Novel Way to Measure and Predict Development: A Heuristic Approach to Facilitate the Early Detection of Neurodevelopmental Disorders

**DOI:** 10.1007/s11910-017-0748-8

**Published:** 2017-04-08

**Authors:** Peter B . Marschik, Florian B. Pokorny, Robert Peharz, Dajie Zhang, Jonathan O’Muircheartaigh, Herbert Roeyers, Sven Bölte, Alicia J. Spittle, Berndt Urlesberger, Björn Schuller, Luise Poustka, Sally Ozonoff, Franz Pernkopf, Thomas Pock, Kristiina Tammimies, Christian Enzinger, Magdalena Krieber, Iris Tomantschger, Katrin D. Bartl-Pokorny, Jeff Sigafoos, Laura Roche, Gianluca Esposito, Markus Gugatschka, Karin Nielsen-Saines, Christa Einspieler, Walter E. Kaufmann, The BEE-PRI Study Group

**Affiliations:** 1 Research Unit iDN—interdisciplinary Developmental Neuroscience, Institute of Physiology, Center for Physiological Medicine, Medical University of Graz, Harrachgasse 21/5, 8010 Graz, Austria; 2 Center of Neurodevelopmental Disorders (KIND), Department of Women’s and Children’s Health, Karolinska Institutet, Stockholm, Sweden; 3 BEE-PRI: Brain, Ears & Eyes—Pattern Recognition Initiative, BioTechMed-Graz, Graz, Austria; 4 Machine Intelligence & Signal Processing group, MMK, Technische Universität München, Munich, Germany; 5 Department of Neuroimaging, Institute of Psychiatry, Psychology and Neuroscience, King’s College London, London, UK; 6 Centre for the Developing Brain, Division of Imaging Sciences and Biomedical Engineering, St. Thomas’ Hospital, King’s College London, London, UK; 7 Department of Experimental-Clinical and Health Psychology, Ghent University, Ghent, Belgium; 8 Child and Adolescent Psychiatry, Center of Psychiatry Research, Stockholm County Council, Stockholm, Sweden; 9 University of Melbourne, Melbourne, Australia; 10 Murdoch Childrens Research Institute, Melbourne, Australia; 11 The Royal Women’s Hospital, Melbourne, Australia; 12 Division of Neonatology, Department of Pediatrics and Adolescence Medicine, Medical University of Graz, Graz, Austria; 13 Chair of Complex and Intelligent Systems, University of Passau, Passau, Germany; 14 Machine Learning Group, Imperial College London, London, UK; 15 Department of Child and Adolescent Psychiatry, Medical University of Vienna, Vienna, Austria; 16 MIND Institute, Davis Health System, University of California, Sacramento, CA USA; 17 Signal Processing and Speech Communication Laboratory, Graz University of Technology, Graz, Austria; 18 Institute for Computer Graphics and Vision, Graz University of Technology, Graz, Austria; 19 Department of Neurology and Division of Neuroradiology, Vascular & Interventional Radiology, Department of Radiology, Medical University of Graz, Graz, Austria; 20 School of Education, Victoria University of Wellington, Wellington, New Zealand; 21 Social & Affective Neuroscience Lab, Division of Psychology—HSS, Nanyang Technological University, Singapore, Singapore; 22 Affiliative Behaviour and Physiology Lab, Department of Psychology and Cognitive Science, University of Trento, Trento, Italy; 23 Department of Phoniatrics, Medical University of Graz, Graz, Austria; 24 Division of Infectious Diseases, David Geffen School of Medicine, University of California, Los Angeles, CA USA; 25 Center for Translational Research, Greenwood Genetic Center, Greenwood, SC USA; 26 Department of Neurology, Boston Children’s Hospital and Harvard Medical School, Boston, MA USA

**Keywords:** Computer vision, Diagnosis, Early human development, Intelligent vocalisation analysis, Multidimensional assessment, Neurodevelopmental disorders

## Abstract

**Purpose of Review:**

Substantial research exists focusing on the various aspects and domains of early human development. However, there is a clear blind spot in early postnatal development when dealing with neurodevelopmental disorders, especially those that manifest themselves clinically only in late infancy or even in childhood.

**Recent Findings:**

This early developmental period may represent an important timeframe to study these disorders but has historically received far less research attention. We believe that only a comprehensive interdisciplinary approach will enable us to detect and delineate specific parameters for specific neurodevelopmental disorders at a very early age to improve early detection/diagnosis, enable prospective studies and eventually facilitate randomised trials of early intervention.

**Summary:**

In this article, we propose a dynamic framework for characterising neurofunctional biomarkers associated with specific disorders in the development of infants and children. We have named this automated detection ‘Fingerprint Model’, suggesting one possible approach to accurately and early identify neurodevelopmental disorders.

## Introduction

Early human development has attracted increasing attention from researchers across scientific disciplines in recent years. This expansion has been driven, in part, by the acknowledgement of the great diversity of (neuro)developmental disorders, their large genetic and phenotypic heterogeneity and the clear need to better understand similarities and differences across syndromes, disorders and disease processes. A continuing series of new paradigms on the functional development of the young nervous system has resulted in progressive changes in approaching typical and atypical early development.

Research on early development during the last decade, in particular, has benefitted greatly from adapting approaches and theories from not only closely related disciplines, such as developmental neuroscience, but also from novel technical and computational fields (e.g. computer vision, machine learning, signal processing, speech and voice analysis). These recent trends are moving towards the disappearance of classic discipline boundaries in investigating early human development and the specificities of various disorders [1, 2].

In this overview—and perspective—paper, we propose a research model aimed at providing (i) a framework for age-specific cross-syndrome comparisons and (ii) eventual prediction of neurodevelopmental outcome. This model may allow for the recognition of syndrome-specific developmental traits and provide an opportunity for earlier identification of disorders, often recognised^1^ at toddlerhood or even later. These particular conditions shall henceforth be referred to as ‘conditions of interest’ (COI), and include for example autism spectrum disorder (ASD), attention deficit hyperactivity disorder (ADHD), Rett syndrome (RTT) and fragile X syndrome (FXS). COI will also apply to infants who have suffered potential deleterious environmental exposures such as, in utero exposure to mosquito-borne diseases like Zika virus (ZIKV), malaria or exposure to sexually transmitted maternal diseases, teratogenic compounds and/or maternal substance abuse.

### Ontogenetic Adaptation

The concept of ontogenetic adaptation and continuity of neural functions [1, 3, 4] plays a central role in our interdisciplinary scientific approach to study infant development. Physiologically speaking, life ex utero as compared to in utero needs to adapt to dramatically different environments and requirements. For survival, it is essential that newborns immediately adapt to their new environment and be embedded within a system that efficiently meets their needs (endogenous or exogenous system perturbations/adaptations; e.g. respiration, nutrition). From our perspective, the most significant changes during this period of development occur in the nervous system, which undergoes the most dramatic, almost permanent adaptation and optimisation during the early postnatal years [1, 3, 5].

Compared to non-human primates, and factoring out vital-functions, we are far less equipped for instant adaptation to the extra uterine life [5–7]. As a consequence, the newborn human is ‘by no means the competent individual which has been sometimes proposed’ [1: 837]. A series of studies on early human postnatal development have indicated that the first 2 months after term are, to a certain extent, a continuity of foetal behaviour [1, 4, 8–10]. Around the end of the second month of life, a major transformation sets in (the 3-month-transformation [4, 5]) and many neural functions change or (gradually) occur leading to a neurobehavioural adaptation to the requirements of extra uterine life. This seems to be specific to the human species and even though we know a great deal about development, the period of the first few months of life is not yet fully understood. However, what we do understand is that this transformation can be characterised by a number of neurofunctional changes, such as an increase in muscle power, postural changes [11, 12], a change in the sucking pattern [13], development of focused visual attention and binocular vision [14, 15] and the beginning of social smiling and cooing vocalisations [16–18].

### Neurological Underpinnings of Behavioural Changes During Infancy

Focusing initially on brain development, the early postnatal period is an intense phase of structured growth and expansion [19–21]. Anatomical changes follow a predictable course in the typically developing brain, when measured using non-invasive cerebral magnetic resonance imaging (MRI), and seem to follow a sigmoidal growth pattern in terms of brain volume [22] as well as white matter structure and content [23, 24]. Functional neuroimaging techniques, such as positron emission tomography (PET), demonstrate concomitant increases in glucose utilisation at around 2 to 3 months, particularly in the parietal, temporal and primary visual cortex, basal ganglia and cerebellar hemispheres. These escalations in metabolism coincide with the emergence of behaviours involving visuospatial and visuo-sensorimotor integration, disappearance or reorganisation of subcortical neonatal behaviours and evidence of increasing cortical activities [25].

At typical term birth, inter-neuronal axonal connections are largely unmyelinated and dendritic sprouting and synaptogenesis is still ongoing. Though the white matter architecture underlying brain connectivity is largely established by term [26], in the first year of life and especially the first 8 months [27], there is a rapid expansion in the spatial distribution and extent of myelination [23, 28] and dendritic sprouting [29]. Linked to this, ongoing proliferation of synapses leads to peaks of synaptic density at the ages of 6–18 months, after which the rate of synaptic pruning overtakes proliferation and a protracted decline begins [30]. Importantly, the rate of myelination, synaptogenesis and synaptic pruning is regionally specific. Generally, the subcortical, cerebellar and primary sensory areas peak in terms of synaptic density first, their connections are myelinated first, and synaptic pruning begins earlier, when compared with other cortical association areas and in particular with the frontal cortex.

What remains unclear is the extent to how these changes associate with cognitive and neurofunctional/behavioural development. It is tempting to assume that behavioural development maps easily onto the maturation profile of the brain itself (e.g. motor and sensory abilities first, then higher-order cognitive functions). Longitudinal studies evaluating MRI at term age and follow-up with neuromotor, behavioural and cognitive assessments during later childhood have also shown links between anatomical features from MRI and later outcome [31, 32]. Furthermore, individual differences in brain growth trajectories have been associated with individual differences in cognitive development at the same age [33]. In all three studies, using three different MRI modalities, features in the subcortical and especially thalamic-associated white matter were the consistent areas that predicted later, or current, language ability and developmental quotient (e.g. areas that mature earlier). It should be noted that only referring to synaptogenesis and myelination is an oversimplification of the complex neurodevelopmental processes, given the developmental changes of neurotransmitter modulation, neuronal differentiation, cortico-cortical connectivity, glia development, etc. that are also taking place.

Given these overlapping early brain processes, it is perhaps not surprising that studying the early brain (before 12 months) has become a larger focus for understanding COI [34–36].

### Developmental Domains of Interest

Development can be seen as a complex autopoietic system, but to better understand the functionality and deviances within it, we need to highlight specific features of this system. Aspects of two major developmental domains (motor- and speech-language) during the first months of life are outlined. Within the scope of this paper, general movements (GMs) and early vocalisations are discussed with respect to both precursors/prerequisites for further development and potential early indicators of neurodevelopmental disorders.

#### Central Pattern Generated Spontaneous Motor Behaviour: The General Movements

Embryonic, foetal and neonatal movement patterns all share characteristics of being endogenously generated. Without being triggered by a specific sensory input, the foetal and neonatal nervous system generates a variety of motor patterns such as startles, general movements, breathing movements, stretching, yawning, sucking, side-to-side movements of the head (rooting) or eye movements [9]. These movement patterns are generated by specific neural networks, the central pattern generators (CPGs), which are located in the brain stem [1, 8, 9, 37]. Some CPGs operate continuously (e.g. respiration), whereas others are activated to perform specific tasks (e.g. sucking, locomotion). In order to lend variability to the motor output, supraspinal projections activate, inhibit and most importantly modulate the CPG activity as does sensory feedback [38•, 39].

The fact that CPG activity was, and partly still is, either overlooked or misinterpreted as reflexes (or even worse as ‘primitive’ reflexes although there is nothing primitive in the developing nervous system) stems from classical neurophysiology. At the end of the 19th century, foetal and neonatal movements were recognised as spontaneously generated; however, reflexology and particularly behaviouristic interpretations tended to ignore these observations [1, 9]. Sir Charles Sherrington studied the contact between the afferent and efferent arch in the spinal cord and introduced experimental lesions to the nervous system in order to eliminate the ‘nuisance’ of spontaneous neural activity. In this way, the relation between stimulus and reflex was extremely consistent as it was not interfered with fluctuations caused by spontaneously generated activity. Although Sherrington [40] himself was fully aware of the artificial nature of his findings and even mentioned that the simple reflex is a fiction, his followers seemed to have ignored this cautionary note and made the reflex pattern the crucial element of neural functions [1, 8, 9].

During the last 20 years of infant studies, attention has shifted from exclusively testing reflexes to additionally assessing spontaneous movements. From the rich repertoire of distinct spontaneous movement patterns to emerge during infancy, the so-called GMs are the most frequently occurring and most complex. During preterm and term age, GMs involve the entire body and manifest themselves in a variable sequence of arm, leg, neck and trunk movements. At a post-term age of 3–5 months, GMs appear as fidgety movements, which are small movements of the neck, trunk and limbs in all directions with variable acceleration [8, 10, 37, 41]. The presence of normal GMs is indicative of normal neurological development, whereas abnormal, monotonous GMs point to neurological deficits. Specifically, the absence of fidgety movements (at 3–5 months) is typically associated with the development of cerebral palsy [37, 41, 42]. In addition to its application in infants with perinatal brain injury, the general movement assessment (GMA) has also been applied to—for example—intrauterine HIV-exposed and/or HIV-infected newborns and young infants [43], infants of mothers with ZIKV infection [44], infants with metabolic disorders [45], infants with genetic disorders [46–49] and infants with ASD [50•, 51].

GMA is not only non-intrusive and cost effective but also has repeatedly proven to be an accurate and reliable assessment tool [41]. In a recent review, Bosanquet and colleagues [42] reported summary estimates of sensitivity and specificity between 98 and 91%, respectively, for the prediction of cerebral palsy. Currently GMA is being used by an increasing number of health professionals around the world for assessment and identification of infants at high-risk for neurological impairments. With this in mind, we are developing a mobile solution allowing for broader application (the GMApp; www.gmapp.idn-research.org; [52]). More recently, complementary efforts have been taken to augment classic GMA, based on visual Gestalt perception, with computer-based movement assessment tools to perform quantitative analysis of GMs [53–57, 58•].

#### Early Vocal Development

In addition to the production of vegetative sounds, infant vocal development during the first weeks of life is mainly characterised by the generation of distress vocalisations, i.e. crying or fussing sounds [59]. At the same time, though a relatively small proportion, the first vowel-like/quasi-vowel non-distress sounds emerge. Most of these sounds do not excite the vocal tract’s full resonance yet and phonation does not involve a distinct systematic mouth opening [17]. These very first vocalisations have also been discussed to be CPG-generated behaviour [60]. Basic understanding of the CPG circuitry for mouth movements and respiration suggests multiple foci in the brain stem [61]. The increasing level of cortical control over sound production leads to the emergence of a more complex type of vocalisation which is representative for the period around 3 months of age, the cooing sound. Often, cooing sounds develop alongside the onset of social smiling in situations of face-to-face interactions [59, 62]. Cooing sounds are (often velar) consonant-like elements, such as voiced fricatives, optionally combined with vowel-like segments [17, 18] with distinct melodic contour [63] representing the first forms of syllables [64]. These sounds vary substantially in structure, quality and temporal organisation as infants certainly have not yet reached full phonetic competence [17, 62]. The production of cooing sounds represents an important step in early vocal development, as for the first time, the emergence of discernible tongue movements, required for typical phonation, becomes apparent [62].

Around the 4th–5th months of age the infant starts to explore the full potential of his/her vocal apparatus and to expand the vocal repertoire by generating sounds with modulations/variations in melody/pitch, loudness and vocal register. These sounds include raspberry vocalisations, squealing, vowel-like elements and more complex marginal babbling (i.e. first slow and shaky transitions between consonant-like and vowel-like sounds). [17, 59, 63]

Deviations from typical early vocal development have been discussed as potential early signs of developmental disorders with a mean age of diagnosis at or beyond toddlerhood (i.e. COI introduced previously). For instance, Patten et al. [65] reported a late onset and low volubility of canonical babbling in infants later diagnosed with ASD. A limited number of studies have focussed on the transition from marginal babbling to canonical or variegated babbling and first word production in neurodevelopmental disorders, with the majority of these studies focussed on volubility measures. Fewer studies have analysed early atypicalities in vocalisation quality measurable by means of acoustic signal level parameters. Furthermore, the multitude of these investigations has been limited to specific vocalisation types, in many cases crying vocalisations, and to a small number of acoustic parameters such as fundamental frequency or (cry) duration [66–68]. In promising pilot work, we demonstrated the potential of more detailed acoustic early vocalisation information retrieval for the identification of infants later diagnosed with RTT [69, 70, 71•, 72].

### Developmental Disabilities with a Late Manifestation or Clinical Onset: A Blind Spot

To study early human development and anticipate neurodevelopmental outcomes, key aspects such as the presence and development of distinctive physical and neurological features and potential neurobehavioural and psychopathological abnormalities are in the focus of interest. Intensive research on genetic disorders, for example, have revealed that certain physical features are associated with specific disorders. In some disorders, these features can be apparent before or immediately after birth (e.g. facial dysmorphia in Down syndrome) and they may also be accompanied by early functional abnormalities that enable an early and accurate diagnosis (e.g. atypical vocalisations in Cri du Chat syndrome). As previously mentioned, there are a number of conditions with no apparent physical features at birth (e.g. ASD, FXS, RTT) and it is only during later development that they reach a saliency threshold leading to a characteristic appearance which contributes to accurate diagnosis [48, 51, 70, 73–76]. When dealing with COI, such as ASD, FXS or RTT for example [73, 77, 78], a formal diagnosis is often given at or beyond toddlerhood. However, parents often raise concerns relating to their child’s delayed or atypical development long before diagnosis [79, 80]. This can lead to substantial difficulties due to long periods of uncertainty where they often follow the path of a diagnostic odyssey.

Let us take FXS as an example. It is the most prevalent form of inherited intellectual disability and one of the most widespread genetic causes of ASD [81, 82]: The majority of clinical features may not be detected or not associated with FXS at an early stage, and thus genetic testing and formal diagnoses are often delayed until, on average, the preschool years [83]. This delay in diagnosis has significant implications for access to early intervention programmes, and results in additional costs and frustration for families. Further, it is not uncommon for additional children to be born with FXS during this time [83]. To remain undetected beyond toddlerhood is not unique to FXS; the same is true for a number of COI that need to be studied in more detail.

A late diagnosis reduces the possibility to study early development and thus hampers research on very young individuals with ‘late’ recognised conditions. Whilst a large number of studies have relied on retrospective parental questionnaires, there has been increasing doubt about the validity of these investigations [79, 84•]. For certain assessments, retrospective video analysis (RVA) is considered to be a more objective method to circumvent the risk of memory bias [84•, 85, 86]. Besides, there has been a lot of effort into prospective studies analysing high-risk populations, e.g. studying infants with an older sibling with ASD or ADHD and prospective studies of infants with single or multiple exposure to intrauterine hazards [87, 88•, 89•, 90–92]. Prospective studies of infants at high risk for ASD, for example, use serial longitudinal direct assessments of behaviour to investigate timing and patterns of symptom onset. These studies have demonstrated that neurofunctional abnormalities may occur even earlier than parents report, in the later half of the first year of life, and may follow a declining pattern, in which infants transition from typical to atypical developmental trajectories over the course of several months.

It goes without saying that a transition from purely CPG-generated activities (e.g. GMs, early vocalisations) into more intentionally controlled behaviours (e.g. movements towards the midline and reaching; modulation in pleasure vocalisations increasingly associated with looking towards the caregiver) gradually evolves with the coexistence of both early spontaneous behaviours and intentionally driven behaviours [4, 10].

## A Novel Approach: The Fingerprint Model

Constantly adapting and optimising neurological functions need to be seen and understood in their development and complexity, i.e. we need to aim for the big picture whilst avoiding the hazards of oversimplification. We need to account for age-specificity when examining children, keeping in mind that this can account for different ‘vulnerabilities’ and the consequential effects for early intervention efficacy [1]. Building upon a concept devised by Prechtl to assess the integrity of the nervous system, it is crucial to (i) take into account the age-specific properties of the nervous system; (ii) to avoid the artificial fragmentation of performances or signs; (iii) to indicate defects but also delays and (iv) take into account the behavioural state of the foetus/infant/child [93].

The ultimate aim of our proposed approach—combined with other biological methods and markers—is to earlier detect suboptimal and pathological development, and, as mentioned at the beginning, achieve a ‘find-early-and-intervene-early’ reality to improve developmental outcomes and avoid diagnostic odyssey.

**Fig. 1:**
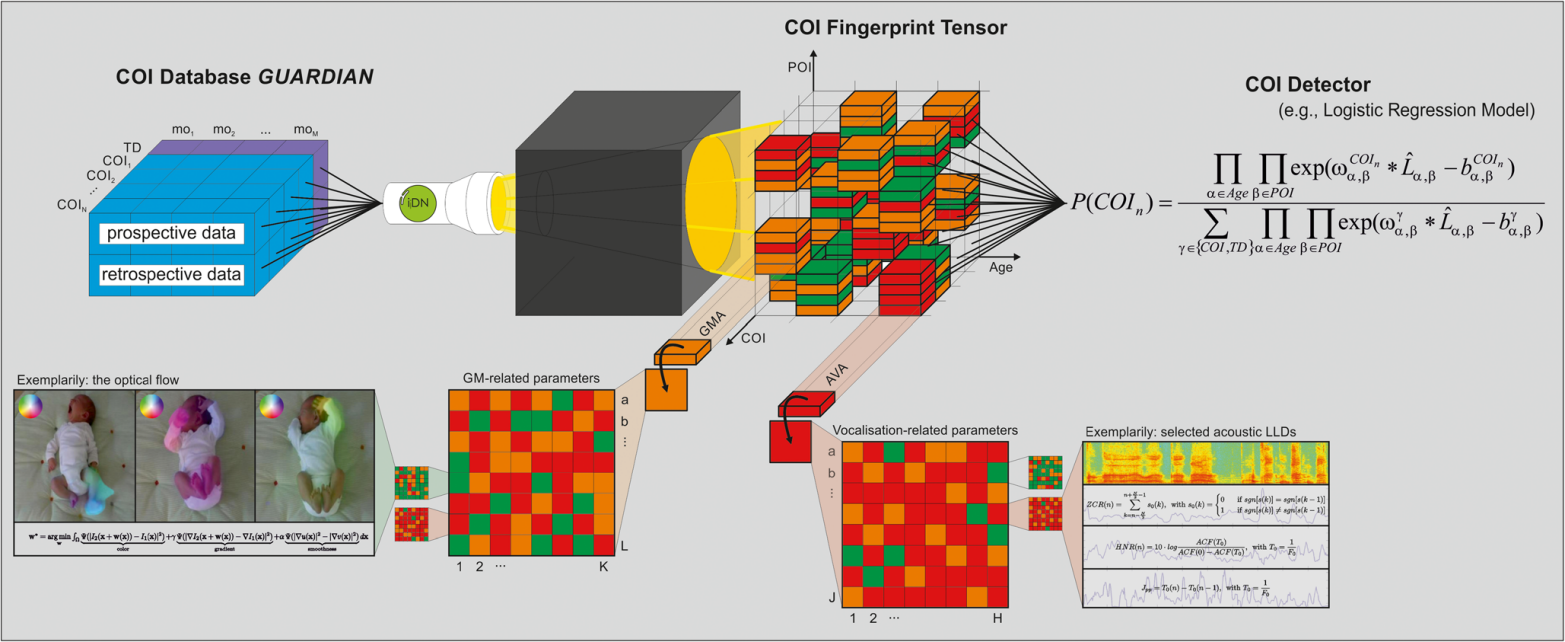
Proposed ‘iDN Fingerprint Model’ for the earlier detection of COI. Our goal is to unravel the early fingerprint of various COI as age-specific POI constellations in an extensive knowledge tensor. Thereby, fingerprint information is modelled in terms of atypicality in objective parameters from POI-related approaches (e.g. GMA in motor development, or AVA of cooing sounds in speech-language development) and underlying levels of representation (e.g. state-of-the-art signal attributes used in audio/video analysis, such as optical flow [94–96], zero-crossings rate, harmonics-to-noise ratio, jitter [97]). Finally, we propose the implementation of a probabilistic model to automatically detect COI from multidimensional data for future clinical application, e.g. by means of logistic regression [98]. *AVA* acoustic vocalisation analysis, *COI* condition of interest, *GM* general movement, *GMA* general movement assessment, *GUARDIAN* Graz University Audiovisual Research Database for the Interdisciplinary Analysis of Neurodevelopment, *mo* month, *POI* parameter of interest, *TD* typical development, Colour code: *green* optimal/normal, *orange* suboptimal, but within the range of normality, *red* atypical)

**Fig. 2:**
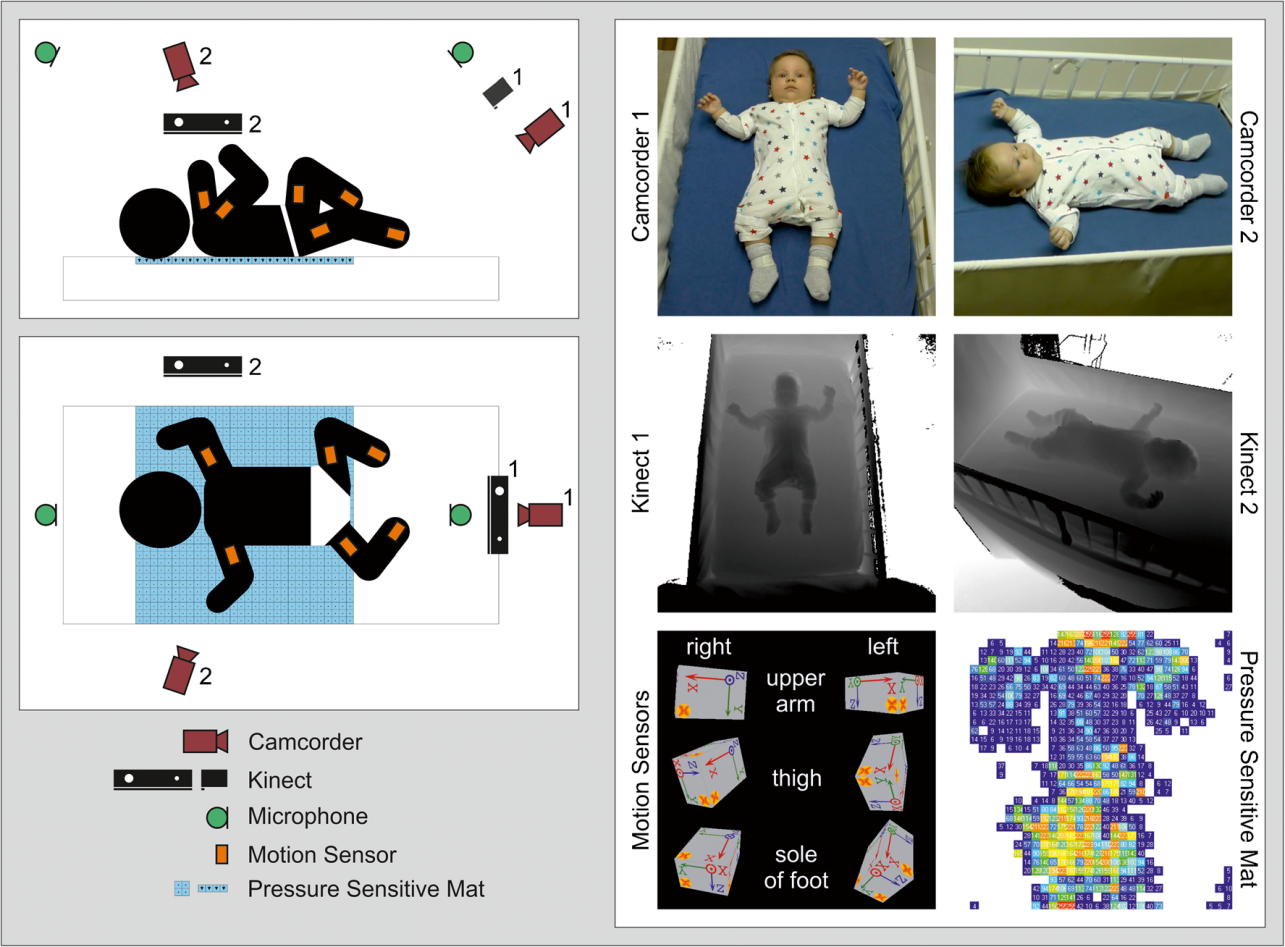
Illustration of a multi-device infant recording setup (schematic on the *left*; sensor view on the *right*): two HD video recordings from different angles (*top right*), two Kinect recordings from different angles (*middle right*), motion tracking (*bottom left*) and contact pressure distribution measurement (*bottom right*) when lying in supine position

To achieve this, we propose a heuristic and probabilistic ‘Fingerprint Model’ (Fig. 1). Such a model requires large age-specific datasets from various developmental domains of typically developing infants and infants with COI. One may however consider this model in the light of neuroconstructivism as an autopoietic model that is already functional whilst it is ‘growing’ [99]. It specifically adopts a maximum likelihood approach whereby age-specific parameters of interest (POI, such as fidgety general movements, one aspect of the motor domain at around 3 months of age; Fig. 2) are used to reliably differentiate between specific COI. Although optimal performance of the model relies upon large and accurate datasets spanning specific age ranges and neurofunctional domains, it can still be effective in providing accurate developmental outcome predictions using incomplete POI datasets. Furthermore, this model has the potential for progressive development in the use of additional POI or newly identified biomarkers which may help to improve accuracy levels of detection. Thus, this model has the potential to develop and expand alongside with new developments across various fields in early human development. The proposed model will encompass analyses at multiple age ranges and include atypical trajectories to detect developmental delays in addition to developmental deviations from typical development [100].

It remains open whether we will be able to define syndrome specific constellations in early infancy or rather identify general signs of atypical development. It is unlikely there will be one signature behaviour in early infancy but rather symptom constellations that pinpoint a certain disorder or subset of disorder (i.e. a fingerprint, an age-specific POI constellation). This approach need not be restricted to neurofunctional assessments but rather be combined with classical biomarker research (most likely a multimarker panel of biomarkers generated from different levels of biological analysis; [101•, 102, 103]). This approach provides a likelihood model that precedes clinical and genetic testing to verify a given suspicion (derived from the model). Our proposed Fingerprint Model should be seen as suggestion or one possibility of an input-output system that allows the detection of certain COI. For the time being, it is as a combined retrospective and prospective approach, with the future aim to prospectively apply certain measures routinely.

## Multidimensional Assessment of CPG-Related Neurofunctions: Preliminary Studies

In order to better understand the 3-month-transformation, in particular, and to define a set of POI for the Fingerprint Model, we are currently conducting a prospective longitudinal study with low-risk infants. Each infant is assessed seven times within their first 4 months of life, with the first assessment taking place at 28 ± 2 days post-term age and subsequent evaluations every 2 weeks, with the last one at 112 ± 2 days. Inclusion criteria of the study are as follows: uneventful pregnancy, uneventful delivery at term age, singleton birth, appropriate birth weight and uneventful neonatal period.

Each assessment session consists of the following three modules: (i) multi-device recordings of endogenously generated neurofunctions; (ii) eye-tracking assessment of visual attention and (iii) video recording of non-nutritive sucking patterns. Here, we highlight the assessment of age-specific spontaneous behaviour in the motor and speech-language domains.

In the assessment module—previously introduced as (i)—the infant is placed in supine position in a standard cot and recorded for 5 min, using the following equipment: (a) camera system: two standard HD camcorders and two Microsoft Kinects; (b) audio recording system: one stereo audio recording device with an additional external studio microphone; (c) motion sensors: six MTw motion sensors attached to the upper arms, upper legs and feet and (d) pressure sensor: one pressure-sensitive mat consisting of a 32 × 32 array of pressure sensels. Figure 2 illustrates the proposed setup. For synchronisation, a clapperboard is placed on the pressure-sensitive mat, with one motion sensor attached to the clapperboard’s clapstick. This generates an easily detectable signal on all employed devices. We perform the synchronisation signal directly before and after the assessment; these two synchronisation signals allow to define a common offset and to compensate a linear clock drift during the measurement.

**Fig. 3:**
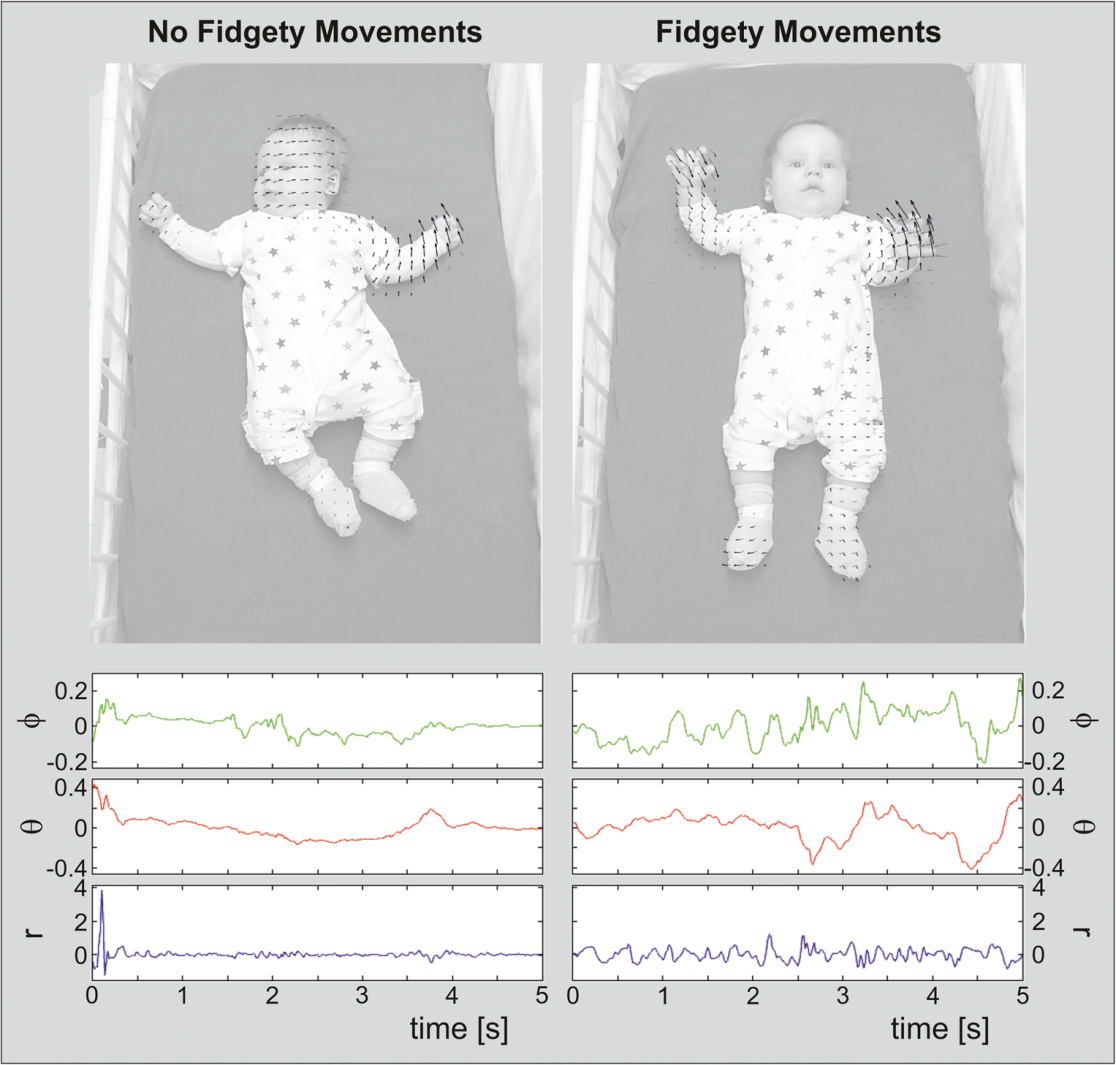
*Top* image frames extracted from video recordings of a male infant, together with the large-displacement optical flow [94, 95]. *Left* infant at 69 days post-term, showing not yet fidgety movements. *Right* corrected age of 84 days post-term age, performing typical fidgety movements. *Bottom* acceleration measured at the right upper arm of the infant within a time window of 5 s around the frames showing the optical flow. The acceleration vector is represented in spherical coordinates, i.e. radius (r), azimuth (phi) and polar angle (theta). For better readability, the means of r, phi and theta have been removed

**Fig. 4:**
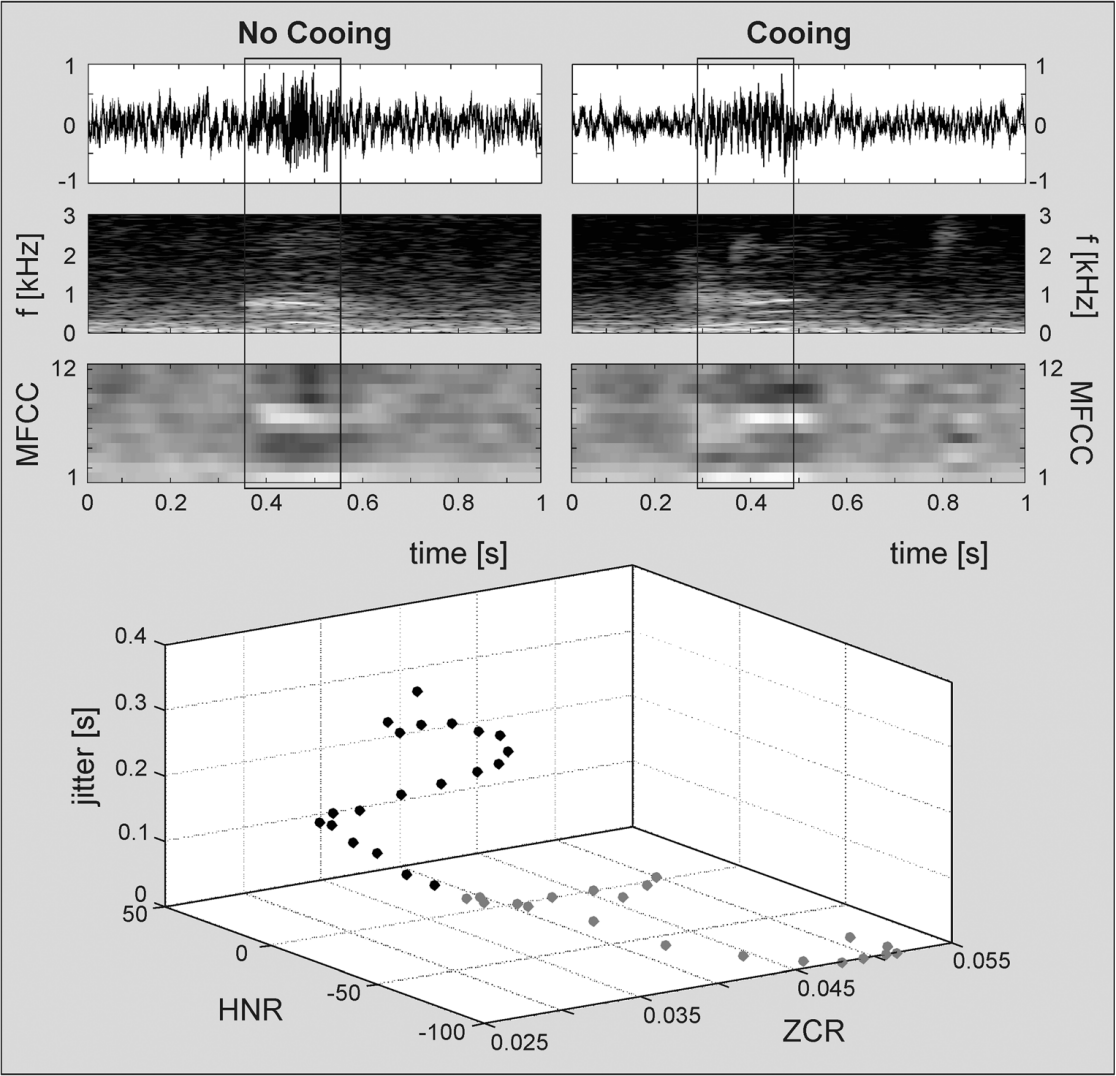
Waveforms, spectrograms and visualised Mel-frequency cepstral coefficients (MFCCs) 1–12 for (*top left*) a vowel-like, low-resonant vocalisation (no cooing), and (*top right*) a typical cooing vocalisation of a female infant at 56 days post-term. The 3D scatter plot (*bottom*) shows the distribution of three exemplarily selected acoustic parameters (mean zero-crossings rate [ZCR], mean logarithmic harmonics-to-noise ratio [HNR], mean local jitter) over 20 frames of 0.01 s extracted from the voiced periods (marked with rectangular boxes in upper plots) of either vocalisation (no cooing: 0.35–0.55 s, *grey dots*; cooing: 0.29–0.49 s, *black dots*)

For these multidimensional recordings, we apply automatic assessments using machine learning. The proposed approach is based on the automated detection of variability of CPG-related functions at signal level. The collected datasets serve as a starting point for our exploratory work on machine learning-based approaches for assessment of spontaneous neurofunctions. Preliminary data obtained (single case differentiation outlined in Figs. 3 and 4) demonstrate the feasibility of our approach. Two specific options, assessment of GMs and assessment of cooing vocalisations, are outlined in the following subsections.

### Automatic GM Assessment

Researching automatic assessment of GMs has received considerable attention in recent years, including computer vision- and motion sensor-based approaches [53–57, 58•].

Within computer vision-based approaches, the use of Kinect sensors is a particularly popular avenue, as they provide in-depth data at a reasonable price. Besides RGB and depth streams (RGB-D), the Kinect devices can also deliver a kinematic stick model of persons, potentially useful for the automatic assessment of infants’ spontaneous motor behaviour. For instance, the motion features that distinguish children at risk for cerebral palsy, as computed by Meinecke and colleagues [104], could be directly applied without the need to use an expensive and complex motion-tracking system. Moreover, the temporal kinematic information could serve as input to advanced machine learning methods, e.g. recurrent neural networks and dynamic Bayesian networks, in order to assess the infants’ motion patterns. However, the kinematic body tracker cannot be applied to individuals shorter than 1 m, since the implemented algorithm was trained on synthetic depth images of avatars ranging from preschool children to adults [105]. Therefore, there is a need to develop kinematic detection and tracking algorithms applicable to infants. Olsen et al. [106] proposed to fit an infant stick model by searching the extreme points in the point cloud corresponding to the depth data.

We aim to follow two alternative directions. The first is to use a similar approach as the original Kinect algorithm [105], i.e. to perform pixel-wise classification of the depth image. For this task, we will need to produce a dataset of synthetic depth images of animated infant body models. Whilst Shotton et al. [105] had a large database of simulated data available, which was rendered with a variety of 3D avatars, our challenge will be to animate the infant models sufficiently in order to cover the whole range of movement. The original Kinect data will serve as test data, which will be manually labelled. The alternative approach will be to use this manually labelled data as training data. For automatic movement assessment, we lack strict real-time requirements that allow us to use more powerful models than the random forest classifier employed by Shotton and colleagues [105], such as convolutional neural networks [107].

In addition to using depth measurements, the state-of-the-art tool for motion analysis in RGB data is based on the optical flow [94, 95], a vector field which measures the movement of image points between two consecutive video frames. The optical flow is often used as input for more advanced motion analysis techniques, e.g. motion-based image segmentation [108], and has also been applied to movement analysis in infants [54].

The alternative to vision-based approaches is the use of motion sensors, comprising accelerometers, gyroscopes and electromagnetic motion trackers. These systems have been applied to GMA in previous studies [53–55, 57, 58•]. Similar to vision-based approaches, motion sensors can also be used to derive a kinematic motion model. A promising avenue might be to combine a vision-based approach with motions sensors, with the possibility of incorporating a pressure device, as suggested previously.

For example, Fig. 3 illustrates the large-displacement optical flows [94, 95], extracted from videos of an infant at 69 days post-term showing no fidgety movements, and later at 84 days post-term performing typical fidgety movements. The current optical flow is illustrated using black arrows. The optical flow extracted 100 ms before this frame is illustrated as grey arrows; this depicts recent changes in 2D velocity, i.e. acceleration. The optical flow in the non-fidgety movement sequence changes rather smoothly over the whole image, whereas in the fidgety movement sequence the optical flow changes its orientation rapidly within different areas of the image. This coincides with the description of fidgety movements as unpredictable and spontaneous movements in all directions [37, 109].

At the bottom of Fig. 3, the acceleration measured at the right upper arm of the infant within a time window of 5 s around the frames in the top of Fig. 3 is illustrated. In both figures, the acceleration vector is represented in spherical coordinates, i.e. radius (r), azimuth (phi) and polar angle (theta). The radius r corresponds to the magnitude of the acceleration, whereas phi and theta correspond to the direction of the acceleration. Compared to the non-fidgety movement sequence, the fidgety movement sequence shows (i) higher short-term variance in r, (ii) larger number of small-to-medium-sized pulses in r and (iii) higher short-term variance in theta and phi, causing many spontaneous changes in the movement direction. Also these characteristics reliably match with Gestalt-based fidgety movements descriptions [37, 109].

Furthermore, instead of following an indirect approach to movement assessment, i.e. estimating a kinematic model first, we can also follow an end-to-end machine learning approach. In end-to-end learning, the recorded data, either from vision systems, motion sensors, or both, is used directly in a machine learning method finding a suitable representation of the data to train the final predictor (classifier or regression model). One current widely used paradigm in machine learning is labelled ‘deep learning’, which is based on (deep) neural networks, stacked restricted Boltzman machines (deep belief networks), stacked auto-encoders, convolutional networks and recurrent neural networks, to name but a few. State-of-the-art feature-based models include support vector machines, decision trees, random forests and probabilistic graphical models [110–115]. When applied to GMA, this will require the manual labelling of the recorded data, for instance the presence, absence or quality of fidgety movements, or the body parts where the movement occurs. Identifying the best combination of sensors to be used and particular learning algorithms appropriate for GMA will be a challenging task in future research.

### Intelligent Early Vocalisation Analysis

In the context of engineering sciences, ‘intelligent’ analysis of data implies a combination of signal processing and machine learning techniques. Applying intelligent audio analysis methodology for early human vocalisation assessment allows the objective identification of early acoustic vocalisation characteristics and potential atypicalities a human listener is incapable of detecting.

Our audio data are segmented manually for infant vocalisations using the multimedia coding system Noldus Observer XT (www.noldus.com). In addition, we are currently developing a system for the automatic detection of infant vocalisations [116]. The segmentation process relies on the definition of infant vocalisations within distinct vocal breathing groups [117]. Pre-linguistic vocalisation types are transcribed according to an annotation scheme adapted from the Stark Assessment of Early Vocal Development-Revised (SAEVD-R; [18]). According to current methods in audio/speech processing [97, 118], a set of acoustic parameters is extracted from each segmented vocalisation by means of the open-source toolkit openSMILE [119, 120]; (www.audeering.com).

Following the ComParE-set of the 2013 to 2017 INTERSPEECH Computational Paralinguistics Challenges, we generate 6373 parameters representing statistical functionals (e.g. arithmetic mean, standard deviation, higher order moments, quartiles) computed for the trajectories of a broad range of acoustic time-, spectral- and/or energy-based short-term low-level descriptors (e.g. spectral band energy, Mel-frequency cepstral coefficients [MFCCs], zero-crossings rate [ZCR], harmonics-to-noise ratio [HNR], jitter) and their derivatives [121, 122]. The acoustic information thereby deduced from the audio recordings constitutes the input to an objective model of early vocal development for successive classification/pattern recognition approaches.

The multidimensional acoustic characterisation of different pre-linguistic vocalisation types (e.g. cooing) facilitates the automatic identification of potential speech-language delays or atypicalities in early vocal development (see Fig. 4).

## Conclusion

Amongst scientists there is a degree of optimism that recent and upcoming (ante portas) technical advancements may shortly reveal new insights into early human development and may provide some clarification with regard to atypical development. Amongst clinicians and parents of children with disabilities however, there is both hope and also scepticism about the foundation of this optimistic view.

The goal of the methodology outlined is to enable the early detection of COI and the opportunity for early intervention. A paradigm shift from a ‘wait-and-see-approach’ to an approach that instead focusses on early identification with the aim to intervene on target (skill) deficits as they emerge is warranted.

We believe that only a comprehensive interdisciplinary approach, combining genetic risk factors with both classical and neurofunctional biomarkers, will enable us to detect and delineate specific parameters for identifying specific neurodevelopmental disorders at a very early age. Cross-syndrome comparisons, including neurotypical subjects, may reveal that some signs are present early in development but have not been detected with current methods. Future will tell whether certain feature constellations pinpoint certain disorders or prove that some disorders naturally unfold beyond this early time window. Besides its limitations and potential shortcomings (e.g. the lack of homogeneous datasets), the proposed ‘iDN Fingerprint Model’ may accurately differentiate abnormal development and specific conditions, as well as reliably predict developmental outcomes.

## Abbreviations

ADHD: attention deficit hyperactivity disorder
ASD: autism spectrum disorder
AVA: acoustic vocalisation analysis
BEE-PRI: Brain, Ears & Eyes—Pattern Recognition Initiative
COI: condition/conditions of interest
CPG: central pattern generator
FXS: fragile X syndrome
GMA: general movement assessment
GMs: general movements
GUARDIAN: Graz University Audiovisual Research Database for the Interdisciplinary Analysis of Neurodevelopment
HNR: harmonics-to-noise ratio
iDN: interdisciplinary Developmental Neuroscience
MFCC: Mel-frequency cepstral coefficient
MTw: wireless motion tracker/trackers
NAS: network-attached storage
POI: parameter/parameters of interest
RTT: Rett syndrome
RGB: red-green-blue colour model
RVA: retrospective video analysis
SAEVD-R: Stark Assessment of Early Vocal Development-Revised
ZIKV: Zika virus
ZCR: zero-crossings rate

## Footnotes

1 ‘Recognised’ refers to a phenotypical onset, clinical manifestation or an accurate (mean age of) diagnosis.
